# Psychological well-being and distress in patients with generalized anxiety disorder: The roles of positive and negative functioning

**DOI:** 10.1371/journal.pone.0225646

**Published:** 2019-11-27

**Authors:** Luca Iani, Rossella Mattea Quinto, Marco Lauriola, Maria Luigia Crosta, Gino Pozzi

**Affiliations:** 1 Department of Human Sciences, European University of Rome, Rome, Italy; 2 Department of Social and Developmental Psychology, Sapienza, University of Rome, Rome, Italy; 3 U.O.C. Psichiatria, Fondazione Policlinico Universitario A. Gemelli IRCCS - Istituto di Psichiatria e Psicologia, Università Cattolica del Sacro Cuore, Rome, Italy; Temple University, UNITED STATES

## Abstract

**Background:**

Whether mindfulness and emotional intelligence may counteract psychological symptoms and whether brooding and worry may be linked to decreased psychological well-being (PWB) in individuals with generalized anxiety disorder (GAD) is still an issue.

**Methods:**

The study used a cross-sectional design on a sample of 66 consecutive individuals with a diagnosis of GAD. Two hierarchical multiple regressions were conducted to determine whether PWB and anxiety symptoms were accounted for by mindfulness and emotional intelligence skills, brooding, and worry.

**Results:**

Worry was negatively related to PWB and showed a tendency to be positively associated with anxiety symptoms after controlling for the other variables. Brooding was uniquely and positively related to anxiety symptoms. Different mindfulness (i.e., describing and nonjudging) and emotional intelligence (i.e., attention and repair) skills were particularly important for PWB. Repair was also negatively related to anxiety symptoms.

**Conclusions:**

Repair was the variable that played a key role in the association with both PWB and GAD symptoms. Worry was the second most important variable, although it approached significance in the relationship with anxiety symptoms. Brooding was more strongly positively associated with anxiety than worry. In sum, the results suggest that an integrated and balanced focus on both positive and negative functioning will be useful in future clinical psychology research to predict, understand, and treat anxiety as well as to examine the antecedents and characteristics of positivity in individuals with GAD and promote their PWB.

## Introduction

Generalized anxiety disorder (GAD) is characterized by excessive anxiety, chronic and persistent worry about different events or activities, and is associated with somatic and psychological symptoms such as restlessness, muscle tension, and sleep disturbance. Clinical characteristics of individuals with GAD also include rumination, which is the tendency to respond to distress by focusing perseveratively on its symptoms and on their possible antecedents and consequences [1].

In recent years there has been increasing recognition of the unsatisfactory degree of remission that current therapeutic strategies entail in GAD [2], paving the way for research on novel approaches to find avenues to understand and treat psychopathological problems of these individuals. For example, introducing measures of positive functioning (e.g., mindfulness, emotional intelligence) into GAD research alongside traditional clinical measures might afford a promising approach [3], because positive functioning constructs have been shown to have a substantial incremental validity in predicting symptoms of psychological disorder [4–5]. Therefore, a better understanding of positive functioning, alongside the assessment of what is commonly studied in GAD research (e.g., worry, rumination), could help to refine treatment and to promote well-being of individuals with GAD.

Worry is a common phenomenon in the general population. However, when it becomes excessive, uncontrollable, and chronically present, it induces emotional discomfort, anhedonia, and disruption [6]. At the cognitive level, excessive worry represents the core diagnostic feature of GAD [7]. Studies showed that worry predicted lower life satisfaction and more negative emotions [8] and was a stronger predictor of anxiety than rumination [9]. However, the above-mentioned studies were conducted with nonclinical samples. Due to the scarcity of studies of positive functioning, it remains unclear whether worry is also linked to decreased psychological well-being (PWB) in individuals with GAD.

Like excessive worry, rumination is repetitive negative thinking as well as a maladaptive emotion regulation strategy that represents a transdiagnostic vulnerability factor in depression and anxiety disorders [10–13]. Unlike worry, rumination stems from an unsuccessful attempt to reach a goal [14]. In particular, brooding is a component of rumination that specifically reflects “a passive comparison of one’s current situation with some unachieved standard” [13].

Research on rumination and brooding has suggested possible associations with well-being and psychological symptoms in GAD. For example, rumination predicted decreases in positive affect in university students [15] and brooding predicted subsequent increases in negative affect in adults [16]. Moreover, both rumination and brooding were moderately positively associated with anxiety symptoms and the relationship between worry and rumination was also moderate [17]. The same study underscored that the positive association of rumination with anxiety remained significant when controlling for depressive symptoms, whereas anxiety was no longer associated with rumination after controlling for worry. Furthermore, individuals with anxiety disorders reported more rumination than nonclinical controls [17]. Finally, the same study suggested that rumination might be more likely found in anxiety disorders in which there is unconstructive repetitive though such as worry. In sum, rumination is an important variable in influencing stress and well-being. However, the association between rumination and positive functioning was mostly based on student samples [15]. It remains unclear whether rumination, or its brooding component, is linked to decreased PWB in individuals with GAD.

In addition to worry and brooding, it is worth examining which aspects of positive functioning may counteract psychological symptoms in GAD. Because individuals with GAD have poor understanding of their emotional states and maladaptive emotional management strategies [18], emotional intelligence seems to provide a promising avenue of investigation. Indeed, emotional intelligence refers to the skills with which individuals attend to their feelings and moods (i.e., attention), experience their feelings clearly (i.e., clarity), and regulate unpleasant moods or prolong pleasant ones (i.e., repair) [19]. Individuals high in emotional clarity experienced a decline in rumination after an experimental stressor [19]. Furthermore, high mood repair predicted a decrease in both depression and anxiety in adolescents, whereas high emotional clarity predicted a decrease in depression only [20]. Moreover, high emotional intelligence predicted both greater life satisfaction and subjective happiness in undergraduate students [21]. Emotional intelligence also mediated the relationship between mindfulness and well-being in university students [22] and the levels of emotional intelligence were low in individuals with various emotional disorders [23]. Interest in emotional intelligence in individuals with GAD has been surprisingly low although the few available studies agreed that mood repair skills were lower in individuals with GAD than in control groups [18, 24].

Another promising avenue of investigation is mindfulness, which can be viewed as a set of skills that incude: labelling internal experiences with words, noticing or attending to external or internal experiences (e.g., sensations, emotions, cognitions), attending to one’s activities of the moment, taking a nonevaluative stance toward thoughts and feelings, and allowing thoughts and feelings to come and go, without becoming absorbed in their content and without getting carried away by them [25]. Different diagnostic groups showed deficits in mindfulness skills [26] and previous research has underscored the need for addressing the role of emotion regulation in the link between mindfulness and well-being [27]. Furthermore, describing internal experiences with words and acting with awareness were important for PWB, especially for individuals with internalizing traits (e.g., augmented stress reactivity and negative affect directed inward) [28]. However, less is known about whether specific aspects of mindfulness are also associated with well-being and anxiety symptoms in individuals with GAD over and above rumination and worry.

There is a need to further investigate if high mindfulness and emotional intelligence skills may be uniquely associated with less anxiety and greater well-being in individuals with GAD. Investigating both positive and negative functioning aspects in a single study may shed light on their specific associations with well-being and anxiety. Moreover, emotional intelligence and mindfulness may have incremental validity over worry and brooding in predicting clinical outcomes, so that not only positive functioning may be associated with less anxiety but also with greater PWB [4].

According to positive clinical psychology, “which has an integrated and equally weighted focus on both positive and negative functioning in all areas of research and practice” [29], we aimed to investigate how mindfulness, emotional intelligence, and PWB were associated with brooding, worry, and anxiety symptoms. Because positive functioning aspects are associated with psychological symptoms [18, 26] and may have incremental validity in predicting clinical outcomes [4], we developed two hypotheses. It was hypothesized that mindfulness and emotional intelligence skills would positively influence PWB after controlling for different aspects of negative functioning (i.e., brooding, worry). It was also hypothesized that mindfulness and emotional intelligence skills would negatively influence anxiety symptoms after controlling for brooding and worry.

## Materials and methods

### Participants and procedure

A total of 66 individuals (20 men and 46 women; *M*_age_ = 42.2 years, *SD* = 12.3) who were attending the outpatient clinic of the Unit of Psychiatry at the Fondazione Policlinico Universitario Agostino Gemelli IRCCS in Rome were interviewed by a psychiatrist during their first visit. Eligible participants had a diagnosis of GAD according to DSM-V criteria, were between 18 and 75 years of age, had no intellectual disability, psychosis, severe personality disorders, or substance abuse problems, had no previous suicide attempts, and had no physical or mental impairments that would prevent them from completing the questionnaires. Written consent was obtained from all participants before data collection. Eighty-three percent of individuals received a primary diagnosis of GAD. Eight percent and 9% of patients received a primary diagnosis of Panic Disorder and a diagnosis that did not fit into either category (e.g., Major Depressive Disorder, Social Anxiety Disorder), respectively. All these patients had a secondary diagnosis of GAD. The study was approved by the Ethical Review Board of Fondazione Policlinico Gemelli (Prot. 33945/16) and was carried out in accordance with the 1964 Helsinki Declaration.

### Measures

Three measures of positive functioning were used. Mindfulness was assessed with the short form of the Five Facet Mindfulness Questionnaire (FFMQ-SF) [30]. The FFMQ-SF measures observing, describing, acting with awareness, nonjudging, and nonreactivity. Higher scores indicate higher levels of mindfulness skills. Cronbach’s αs were .77, .60, .70, .57, and .59 for describing, observing, acting with awareness, nonreactivity, and nonjudging, respectively. The observing, nonreactivity, and nonjudging scales had insufficient reliability. According to Ziegler et al. [31], the omega coefficient would be more suitable than the Cronbach’s α for assessing the reliability of short scales. Indeed, omega coefficients for all mindfulness scales were greater than the widely used rule of thumb of .60 [32] (ω = .79, .61, .75, .61, and .64 for describing, observing, acting with awareness, nonreactivity, and nonjudging, respectively). Emotional intelligence was assessed with the Trait Meta-Mood Scale-24 (TMMS-24) [33] measuring attention, clarity, and repair. Higher scores indicate higher levels of emotional intelligence. Translation and back-translation procedures were used to obtain appropriate meanings for the TMMS-24 questions. The TMMS-24 Cronbach’s αs were .78, .91, and .88 for attention, clarity, and repair, respectively. Psychological well-being was assessed with the 18-item version of the PWB Scales [34] measuring self-acceptance, positive relations with others, autonomy, environmental mastery, purpose in life, and personal growth. Higher scores indicate higher levels of PWB. Cronbach’s α for the PWB total score was .78.

Three measures of negative functioning were used. Brooding was assessed with the Brooding subscale of the Ruminative Responses Scale [13]. Higher scores indicate higher levels of brooding. Cronbach’s α was .77. Worries were assessed with the 8-item version of the Penn State Worry Questionnaire (PSWQ-A) [35]. Higher scores indicate higher levels of worry. Cronbach’s α was .91. Generalized anxiety disorder was assessed with the GAD-7, which is a tool for screening for GAD and assessing its severity [36]. Higher scores indicate higher levels of GAD symptoms. The mean GAD-7 score of the group is within the range for moderate severity (*M* = 11.1; *SD* = 5.4). Cronbach’s α was .89.

Information about comorbidity was obtained from the medical record. Demographic characteristics were collected with questions regarding age, sex, education, marital status, and employment status.

### Data analysis

Associations between variables were evaluated with Pearson’s *r*. Two hierarchical multiple regressions were conducted to determine whether PWB and anxiety symptoms were accounted for by positive and negative functioning aspects. The sequence of independent variables in hierarchical multiple regressions is guided by the purpose and logic of the research, so that these variables may be ordered according to specific questions to be answered by the research [37]. Consistent with the hypotheses of the study, the independent variables were entered in the following order: brooding, worry, mindfulness skills (i.e., observing, describing, acting with awareness, nonjudging, and nonreactivity), and emotional intelligence skills (i.e., attention, repair, and clarity) to examine their associations with both PWB and anxiety symptoms. The diagnostics for multicollinearity revealed acceptable results (all VIF values <2.5). Occasional missing values were imputed by calculating, for each participant, the average score for each subscale and then replaced.

## Results

### Correlations among variables

#### Negative cognitive styles

GAD symptoms and worry had positive correlations with brooding (*r* = .60 and .51, respectively) and negative correlations with repair (*r* = -.44 and -. 45, respectively). GAD symptoms were positively associated with worry (*r* = .56) and negatively associated with PWB (*r* = -.44). Worry was also negatively associated with PWB (*r* = -.54). The correlations of brooding, worry, and GAD symptoms were larger with acting with awareness and nonjudging than the corresponding correlations with describing and observing.

#### Mindfulness and emotional intelligence variables

Describing was positively associated with clarity and PWB (*r* = .64 and .54, respectively). Repair was also positively associated with PWB (*r* = .51). Observing was not associated with any variable, whereas nonreactivity was negatively associated with brooding and GAD symptoms only (*r* = -.25 and -.22, respectively). Table 1 reports the correlations among all variables in the study along with means and standard deviations.

**Table 1:** Relationships between variables.

	Mean	Standard deviation	1	2	3	4	5	6	7	8	9	10	11	12
1. Describing	17.28	4.33	1											
2. Observing	13.16	3.67	.24	1										
3. Acting with awareness	18.48	3.83	.18	.13	1									
4. Nonreactivity	12.48	3.34	.21	.04	-.19	1								
5. Nonjudging	12.95	3.40	.05	.09	.17	.04	1							
6. Attention	27.98	5.43	.00	-.07	-.09	-.08	-.46***	1						
7. Clarity	25.71	6.48	.64***	.15	.00	.09	-.21	.16	1					
8. Repair	23.75	6.24	.37**	.21	.26*	.05	.08	.02	.41***	1				
9. Brooding	13.09	3.50	-.28*	-.18	-.34**	-.25*	-.46***	.39**	-.12	-.35**	1			
10. Worry	28.08	7.21	-.25*	-.18	-.32**	-.13	-.41***	.37*	-.10	-.45***	.51***	1		
11. Psychological well-being	70.66	12.75	.54***	.14	.36**	.04	.10	-.32*	.38**	.51***	-.44***	-.54***	1	
12. GAD symptoms	11.41	5.16	-.20	-.12	-.36**	-.22*	-.32**	.23	.04	-.44***	.60***	.56***	-.44***	1

*Note*. GAD *=* Generalized anxiety disorders.

* p < .05

** *p <* .01

*** *p* < .001.

### Regression analyses

Since we did not find any effect on PWB and anxiety symptoms due to comorbidity (PWB: *t* = .89, *p* = .375; GAD-7: *t* = .02, *p* = .983), age of onset of GAD symptoms (PWB: *t* = .26, *p* = .798; GAD-7: *t* = 1.77, *p* = .085), and whether GAD was the unique diagnosis or not (PWB: *t* = .01, *p* = .994; GAD-7: *t* = -.36, *p* = .722), we excluded these variables from the analyses.

Table 2 reports the final regression models that describe the relationships of positive and negative functioning variables with PWB and anxiety symptoms, respectively (details are provided in S1 and S2 Tables). In the first step of the regression model, brooding was negatively associated with PWB (β = -0.44, *p* < .001) and accounted for 19% of the variance. In the second step, worry revealed a significant negative association with PWB (β = -0.43, *p* < .001) and accounted for an additional 14% of the variance. Introducing the mindfulness skills in the third step explained an additional 21% of the variance, with a significant positive association of describing with PWB (β = 0.41, *p* < .001). Finally, adding the emotional intelligence skills in the fourth step explained an additional 8% of the variance, with attention (β = -0.30, *p* = .006) and repair (β = 0.23, *p* = .042) being significantly negatively and positively associated with PWB, respectively. When all the independent variables were included, worry, describing, nonjudging, attention, and repair were associated with PWB accounting for 61% of the variance.

**Table 2:** Hierarchical regression analysis for variables associated with psychological well-being and anxiety symptoms: Model 4.

		*ΔR*^2^	*β*
Psychological well-being			
Step 1		.19***	
	Brooding		-.12
Step 2		.14***	
	Worry		-.26*
Step 3		.21***	
	Observing		-.09
	Describing		.38**
	Acting with awareness		.11
	Nonjudging		-.24*
	Nonreactivity		-.11
Step 4		.08*	
	Attention		-.30**
	Clarity		.00
	Repair		.23*
Total *R*^2^		.61*	
**GAD**			
Step 1		.36***	
	Brooding		.37**
Step 2		.09**	
	Worry		.24†
Step 3		.03	
	Observing		.03
	Describing		-.10
	Acting with awareness		-.11
	Nonjudging		.04
	Nonreactivity		-.12
Step 4		.07†	
	Attention		-.05
	Clarity		.30*
	Repair		-.26*
Total *R*^2^		.54†	

Note.

†*p <* .10

*p < .05

**p < .01

***p < .001

In the first step of the regression model, brooding was positively associated with GAD symptoms (β = 0.60, *p* < .001) and explained 36% of the variance. In the second step, worry revealed a significant positive association with GAD symptoms (β = 0.35, *p* = .002) and explained an additional 9% of the variance. In the third step, the mindfulness skills were not associated with GAD symptoms (*R*^2^ = .02, *ns*). Finally, adding the emotional intelligence skills in the fourth step explained an additional 7% of the variance, with clarity (β = 0.30, *p* = .034) and repair (β = -0.26, *p* = .038) being significantly positively and negatively associated with GAD symptoms, respectively. When all the independent variables were included, only brooding, clarity, and repair revealed a significant association with GAD symptoms with a slight tendency of worry, accounting for 54% of the variance (Table 2).

Because a problem of content overlap between worry items and anxiety symptoms items may occur, the analysis was repeated after removing items 2 (“Not being able to stop or control worrying”) and 3 (“Worrying too much about different things”) from the GAD-7. This analysis showed similar results (Final model: *R*^2^ = .57; brooding: β = 0.43, *p* = .001; worry: β = 0.25, *p* = .043; clarity: β = 0.29, *p* = .034).

## Discussion

Positive clinical psychology has begun to examine how positive functioning (e.g., well-being, psychological strengths) and negative functioning (e.g., distress, psychological vulnerabilities) are interrelated in mental health. Applying positive psychology principles to the clinical setting may improve the prediction of psychological symptoms, lead to better long-term outcomes of treatment (e.g., long-lasting recovery from mental disorders), and increase resilience and psychological well-being of patients. Indeed, knowing which emotional intelligence and mindfulness skills are more strongly associated with anxiety symptoms and PWB in individuals with GAD can be helpful for the refinement of treatment.

Our findings show that mindfulness and emotional intelligence skills are uniquely associated with PWB and anxiety symptoms. Describing internal experiences with words, attention to feelings, and mood repair were particularly important for PWB of individuals with GAD. Previous research has shown that adolescents with GAD reported a higher rate of alexithymia, which reflects a deficiency in understanding, processing, and describing emotions [38] and is strongly and inversely related to emotional intelligence [39], when compared with control adolescents [40]. Paralleling previous studies with nonclinical samples [41–42], high describing was positively associated with greater PWB in our sample. This result is consistent with a previous research in which describing internal experiences with words positively influenced different well-being outcomes in individuals with internalizing traits (e.g., negative affect directed inward) [28]. Moreover, the tendency to label emotional states with words was found to be positively associated with greater mindful emotion regulation [43], a process that might facilitate the experience of well-being also in GAD.

The degree to which individuals with GAD attended to their moods and emotions was negatively associated with PWB. This result is consistent with previous studies in which low attention to feelings predicted greater positive mood [20] and mental health [44]. The tendency to pay too much attention to one’s emotions, especially when they are negative, is considered a maladaptive strategy that may trigger rumination, which, in turn, may be harmful to well-being. Indeed, also the correlations between brooding and attention were moderate and positive in our sample.

The tendency to believe that one can repair negative moods or maintain positive ones was positively associated with PWB. This result is consistent with previous studies [20, 44]. The degree to which individuals with GAD considered negative mood to be repairable reflects the tendency to use positive thinking to regulate affective states. It is possible that mood repair may allow these individuals to develop adaptive strategies for dealing with negative moods, which, in turn, may enhance their well-being.

The hypothesis that specific mindfulness and emotional intelligence skills would positively influence PWB after controlling for brooding and worry was supported in the present study. At a more general level, this result can be interpreted through the lens of positive clinical psychology. In particular, it suggests that positive functioning may be beneficial for well-being of individuals with GAD.

Only worry was negatively associated with PWB when controlling for other variables, whereas brooding was not. Worry is related to cognitive avoidance of negative thoughts, inhibits the ability to use adaptive coping resources, and serves an emotion-laden imagery avoidance function that is associated with future threats [45]. Indeed, although individuals with GAD who use worry to cope with emotional distress tend to believe they are solving problems related to upcoming threats, worry becomes a dysfunctional coping style because no effective solutions to the problem are available. Pathological worriers tend to have negative expectations about the future, and, consequently, to report lower well-being. The importance of worry for PWB - as evidenced in the present study - is consistent with previous research showing that remitted individuals with anxiety disorders displayed less PWB compared with healthy controls [46]. A different interpretation is that individuals with GAD cannot accept their positive emotional responses, thus diminishing their ability to enjoy and to experience well-being [47]. Brooding was no longer associated with PWB when controlling for other variables. It seems that worry and different positive functioning aspects (e.g., describing with words and attention to feelings) are more strongly associated with PWB in individuals with GAD and tend to be more important than brooding. To our knowledge, this is the first study to show an association of brooding with PWB in individuals with GAD when controlling for other positive and negative functioning aspects.

Brooding was particularly important for anxiety symptoms in individuals with GAD. This result is surprising because previous research has suggested that rumination may affect disorder-specific aspects of depression, whereas high worry is more likely to be linked with disorder-specific aspects of anxiety. Indeed, previous research has shown that only brooding mediated the relationship between negative affectivity and depressive symptoms, whereas only worry mediated the association between negative affectivity and anxiety symptoms [48]. Moreover, worry predicted anxiety after controlling for rumination, whereas rumination was no longer associated with anxiety after controlling for worry [49].

The relevance of brooding for anxiety symptoms in the present study indicates that the tendency to think anxiously or gloomily when comparing the current situation with an unachieved standard leads individuals with GAD to increase anxiety symptoms. This result is consistent with a previous study showing that high brooding uniquely predicted greater anxiety in participants attending a community outpatient mental health clinic for anxiety and affective disorders [50]. In contrast to [17], in our study worry is not the anxiety aspect that drives its relationship with rumination. Instead, ruminative self-awareness seems to be not adaptive for anxious patients also when controlling for worry.

Worry tended to be positively associated with anxiety symptoms. An interpretation of this result is that individuals with GAD, which use pathological worry in an attempt to solve upcoming threats, seem to fail to effectively cope with the threat. This, in turn, leads to unwanted consequences (i.e., anxiety increase) that drive these individuals to continue to use worry as a process of cognitive avoidance, thus reinforcing the vicious cycle [47]. Nevertheless, individuals with anxiety tend to show excessive negative affect, typically in the form of fear, in response to negative events. Moreover, high worry predicted greater anxiety also in nonclinical participants [51]. However, worry had a secondary role in the association with GAD symptoms when controlling for all other variables. In sum, the hypothesis that high brooding and worry would positively influence anxiety symptoms after controlling for mindfulness and emotional intelligence was partially supported in the present study.

High repair was negatively associated with GAD. This result is consistent with previous studies in which high repair was negatively associated with anxiety in individuals with GAD [24]. The result that repair is important for anxiety symptoms is in line with previous studies suggesting that emotional intelligence has the potential to effectively treat emotional avoidance, which is typical in anxiety disorders [52]. Indeed, if individuals with anxiety can accept the unpleasant emotions associated with thinking about a threat image, they should be facilitated in using effective problem-solving strategies and, at the same time, should be less likely to use cognitive avoidance strategies.

Mindfulness skills were not associated with anxiety symptoms. This result is somewhat unexpected, given that MBIs have shown efficacy in reducing anxiety symptom severity in individuals with a variety of disorders [53]. An interpretation of this result could be that brooding and worry completely mediated the effects of mindfulness on anxiety symptoms. This interpretation is also consistent with previous research showing that repetitive negative thinking (i.e., worry and rumination) was a significant and unique mediator of the effects of MBIs on clinical outcomes [54].

### Limitations and future directions

This study has some limitations. First, the generalizability of our findings is limited since this study was based on self-reported cross-sectional data from a relatively small sample. Future prospective or longitudinal studies with larger samples of individuals with GAD would be required to establish causal links between variables and to generalize the results. Second, because we did not assess participants’ meditation experience, our study cannot compare the role of trait mindfulness vs. meditation training on PWB or anxiety symptoms. However, previous research has considered meditation experience as an antecedent of mindfulness skills, showing that the effect of meditation on well-being was fully mediated by improvement in trait mindfulness [55]. Thus, although we did not measure a possible distal factor of PWB, we considered the five proximal factors that can be cultivated through meditation. Third, nonjudging, nonreactivity, and observing had low reliability in the present study. Nevertheless, we think that the modest reliability of these scales might be due to the specific characteristics of the sample. Indeed, the FFMQ-SF was previously validated using a larger nonclinical sample with a significantly higher education level than patients enrolled in our study [28]. Moreover, according to Ziegler et al. [31], the omega coefficients for these scales were acceptable.

Although MBIs were effective in reducing anxiety symptoms and promoting well-being [56–58], this study seems to be the first to examine the extent to which specific aspects of mindfulness and emotional intelligence are associated with well-being and anxiety symptoms in individuals with GAD over and above rumination and worry. As such, it has implications for the promotion of PWB as well as the improvement of anxiety treatment in these individuals. Although MBIs have been shown to reduce symptoms in individuals with GAD [59], less is known about the role of emotional intelligence in promoting PWB and reduce anxiety symptoms in these individuals. If greater ability to be mindful and manage emotions is associated with high levels of well-being and low levels of anxiety, future research could examine whether mindfulness interventions, based on the development of emotional intelligence skills and targeting brooding and worry of individuals with GAD, might lead to positive health. Relating to this point, some recent therapeutic approaches have integrated mindfulness and emotional intelligence to reduce human suffering and promote human functioning [52]. We hypothesize that these approaches, aimed at developing the ability to experience emotions without impeding effective actions (e.g., problem-focused coping), would reduce the suffering of individuals with GAD and promote their PWB. We also think that an integrated and balanced focus on both positive and negative functioning will be useful in future clinical psychology research to predict, understand, and treat anxiety as well as to examine the antecedents and characteristics of positivity in individuals with GAD and promote their PWB.

## Supporting information

S1 Table Hierarchical regression analysis for variables associated with psychological well-being: Models 1-4. (XLSX)

S2 Table Hierarchical regression analysis for variables associated with anxiety symptoms: Models 1-4. (XLSX)
